# Temporal-spatial deciphering mental subtraction in the human brain

**DOI:** 10.1007/s11571-023-09937-z

**Published:** 2023-02-27

**Authors:** Na Clara Pan, Chengtian Zhao, Jialin Du, Qilin Zhou, Cuiping Xu, Chunyan Liu, Tao Yu, Dan Zhang, Yuping Wang

**Affiliations:** 1 Department of Neurology, Xuanwu Hospital, Capital Medical University, No. 45, Changchun Street, Xicheng District, 100053 Beijing, China; 2 Beijing Key Laboratory of Neuromodulation, No. 45, Changchun Street, Xicheng District, 100053 Beijing, China; 3 Department of Neurology, Aviation General Hospital, Courtyard 3, AnwaiBeiyuan, Chaoyang District, 100012 Beijing, China; 4 Department of Pharmacy Phase I Clinical Trial Center, Xuanwu Hospital, Capital Medical University, No. 45, Changchun Street, Xicheng District, 100053 Beijing, China; 5 Beijing Institute of Functional Neurosurgery, Xuanwu Hospital, Capital Medical University, No. 45, Changchun Street, Xicheng District, 100053 Beijing, China; 6 Department of Psychology, Tsinghua University, Haidian District, 100084 Beijing, China; 7 Institute of sleep and consciousness disorders, Center of Epilepsy, Beijing Institute for Brain Disorders, Capital Medical University, Fengtai District, 100069 Beijing, China

**Keywords:** Mental arithmetic, Cognitive control, Electrophysiology, Mismatch detection

## Abstract

Mental subtraction, involving numerical processing and operation, requires a complex interplay among several brain regions. Diverse studies have utilized scalp electroencephalograph, electrocorticogram, or functional magnetic resonance imaging to resolve the structure pattern and functional activity during subtraction operation. However, a high resolution of the spatial-temporal understanding of the neural mechanisms involved in mental subtraction is unavailable. Thus, this study obtained intracranial stereoelectroencephalography recordings from 20 patients with pharmacologically resistant epilepsy. Specifically, two sample-delayed mismatch paradigms of numeric comparison and subtracting results comparison were used to help reveal the time frame of mental subtraction. The brain sub-regions were chronologically screened using the stereoelectroencephalography recording for mental subtraction. The results indicated that the anterior cortex, containing the frontal, insular, and parahippocampous, worked for preparing for mental subtraction; moreover, the posterior cortex, such as parietal, occipital, limbic, and temporal regions, cooperated during subtraction. Especially, the gamma band activities in core regions within the parietal-cingulate-temporal cortices mediated the critical mental subtraction. Overall, this research is the first to describe the spatiotemporal activities underlying mental subtraction in the human brain. It provides a comprehensive insight into the cognitive control activity underlying mental arithmetic.

**Supplementary Information:**

The online version contains supplementary material available at 10.1007/s11571-023-09937-z.

## Introduction

Mental arithmetic is not only a basic daily faculty, but also provides a powerful paradigm for characterizing fundamental cognitive processes (Nieder and Dehaene 2009; Houdé et al. 2010). Prior research has indicated that a low arithmetic attainment is attributed to a deficit in the general cognitive abilities (Bull et al. 2008). Over the last 30 years, cognitive arithmetic has been the focus of extensive experimental research. However, subtraction has received considerably insufficient attention from researchers than addition or multiplication. Especially, the psychological studies focusing on subtraction solving are surprisingly scarce compared with those focusing on addition, probably because the former is often implicitly assumed to be cognitively similar to the latter, its mathematical inverse. Cognitively, adding and subtracting are composed of several different strategies (Fayol and Thevenot 2012). Subtraction problems tend to be solved using more procedural approaches than the addition ones (Campbell and Xue 2001). Therefore, in recent times, understanding the mental substrates and neurocognitive mechanisms of subtraction has been an important line of interdisciplinary research.

Mental subtraction contains different cognitive processes, including numerals recognition, numeric comparison, performing mathematical operations, remembering the results, maintaining attention, and other more specialized processes (Kong et al. 2000; Rickard et al. 2000; Pesenti et al. 2001; Pinel et al. 2001, 2004; Dehaene et al. 2003; Dimitriadis et al. 2010). Functional imaging studies have identified a consistent network of brain regions that are significant for mental subtraction. As compared to addition, the functional magnetic resonance imaging’s (fMRI) findings revealed significantly greater activation during subtraction in regions along the dorsal pathway, including the left inferior frontal gyrus (IFG), the middle portion of the dorsolateral prefrontal cortex, and the supplementary motor area (SMA) (Yang et al. 2017); furthermore, subtraction also had more activation in the postcentral gyrus (postCG), superior temporal gyrus (STG), thalamus (Ni et al. 2011) and precentral gyrus (preCG) (Abd Hamid et al. 2011). Additional data in healthy adults have revealed that the intraparietal sulcus and the posterior superior parietal lobe are more active during subtraction than multiplication (Dehaene et al. 2003); however, the left angular gyri (AG) and supramarginal gyri (SG) were modulated to a greater degree by multiplication than subtraction (Ischebeck et al. 2006). Moreover, different algorithms, such as simple addition, subtraction, and multiplication tasks, have demonstrated common activation in the middle frontal gyrus (MFG), inferior temporal gyrus (MTG) and occipital lobes (Ischebeck et al. 2009; Ni et al. 2011). These imaging data contributed widely to help us understand the cerebral substrates involved in subtraction. However, the dynamic temporal course of mental subtraction with a high temporal resolution in the human brain has been inefficiently documented thus far.

In addition to the fMRI, evoked (phase-locked) and induced (non-phase-locked) activities in the electroencephalography (EEG) pattern have highlighted neurophysiological patterns of mental subtraction in the human brain. The event-related potential (ERP) results were employed to identify several negative components between 200 ms and 400 ms in subtraction that have been referred to as N200, N270, N300, P300 and N400, compared with addition or multiplication (Jasinski and Coch 2012; Taghizadeh et al. 2021; Gao et al. 2022). The event-related desynchronization and synchronization (ERD/ERS) also revealed that subtractions is associated with a lower theta ERS than multiplication in the frontal and parietooccipital cortices (Brunner et al. 2021); additionally, it displayed a higher alpha ERD than addition, with the largest difference in the parietooccipital cortex (De Smedt et al. 2009a). The scalp EEG data offered excellent temporal, however, limited spatial resolution. Therefore, direct evidence regarding the temporospatial cortical activation patterns and mechanisms during mental subtraction remains insufficient.

To explore the neural basis underlying the temporal dynamic process among the different brain patterns during the complete mental subtraction operation, the intracranial stereoelectroencephalography (SEEG) recordings were used to offer a high temporal resolution and precise spatial localization information of the human brain’s cognitive activities. The delayed match/mismatch-to sample (DMS) paradigms were applied to engage the SEEG during early numeric comparison and following mental subtraction operation. In Task 1, we utilized the DMS paradigm of two double-digit numbers to examine incongruity detection, which was contained in the process of numeric comparison (Gómez-Velázquez et al. 2015). Furthermore, the mental subtraction was significantly associated with number comparison (De Smedt et al. 2009b) and relay on this procedure, even sharing an evolutionary neural system involved in numeric comparison (Prado et al. 2011). In Task 2, the DMS paradigm of the mental subtraction results was applied to assess the correctness of mental subtraction. Therefore, both tasks would indicate the time window of the subtraction operation in the brain, that is from the early stage before subtraction operation to result-decision after subtraction. By comparing different conditions in the same task, the spatiotemporal landscape of mental subtraction could be deciphered.

This study attempted to addressed the dynamic neural basis of mental subtraction. A simple course of mental subtraction with few other associated cognition activities, including numeric recognition, task-dependent attention, and result memorization, were abstracted. Furthermore, the mechanism containing both the structural pattern and neural activities during this course of mental subtraction have been described. Our results provided important neural evidence to highlight that the gamma (beta/gamma) band activities in the parietal-limbic-temporal lobes mediated mental subtraction.

## Methods

### Participants

The intracranial recordings were obtained from 20 patients (5 women and 15 men) with pharmacologically resistant epilepsy at the Xuanwu Hospital, Capital Medical University, Beijing, China. Their ages ranged from 13 to 52 years (mean = 25.65, SD = 8.95). All patients were implanted with stereotactic intracranial electrodes for diagnostic purposes as part of their evaluation for the neurosurgical epilepsy treatment. The patients who met strict criteria of inclusion and exclusion could be recruited. Those with abnormal brain structure or destructive lesions, such as tumors or encephalomalacia, were excluded. All patients had normal or corrected-to-normal vision and were right-handed. No clinical seizures occurred during the experiment. The study was approved by the Ethics Committee of the Xuanwu Hospital, Capital Medical University (Project number: [2017]086); further, each patient provided informed consent to participate in the research.

### Experimental task

This study used a delayed match-to-sample paradigm. The visual stimuli consisted of a pair of white Arabic double-digit numbers (ranging from 11 to 49) that were sequentially presented on a black background. The first stimulus (S1) was followed by a smaller, second one (S2). S1 and S2 were presented on the screen for 300 ms each, with an interstimulus interval of 200 ms. The interval between the end of the previous S2 and the onset of the subsequent S1 was 5 s.

The experiment was divided into two tasks. In Task 1, the participants were required to judge whether S2 was identical to S1. It was divided into 2 rounds, each comprising 40 trials. The stimulus pairs were randomized with an equal occurrence rate of the following two conditions: (i) S1 and S2 were identical (S1 = S2); (ii) S1 and S2 were different (S1 ≠ S2). In Task 2, the participants were required to assess whether the difference between S2 and S1 (S2-S1) was 3. Task 2 was also divided into 2 rounds, each comprising 60 trials. The stimulus pairs were randomized with an equal occurrence rate of the following three conditions: (iii) the difference between S2 and S1 was 0 (S1-S2 = 0); (iv) the discrepancy between S2 and S1 was 3 (S1-S2 = 3); (v)the difference between S2 and S1 was unequal to 3 or 0 (S1-S2 ≠ 3/0).

The participants were encouraged to concentrate on the center of the screen and judge the answer to be “YES” or “NO” by pressing the appropriate button on a push pad. They were instructed to respond as quickly and as accurately as possible. The left and right button pressing in each run was counterbalanced. All rounds began with a resting-state period of 3–10 min. The stimuli were presented on a standard liquid crystal display screen using E-Prime software (version 2.0, Psychology Software Tools, PA); the averaged visual angle of the picture was adjusted to 2.1° at ~ 50 cm viewing distance.

### SEEG recordings

The patients were implanted with 0.8-mm diameter SEEG electrodes (Sinovation (Beijing) Medical Technology Co., Ltd., Beijng, China). The depth electrodes were semi-rigid platinum/iridium ones, with contacts lengths that were 2 mm long with 1.5 mm interval distances. Each electrode had 8–18 contacts depending on its length. The SEEG was recorded using the Neuroscan system (Scan 4.5; Neurosoft Labs Inc.) with a 128-channel SynAmps EEG/EP amplifier (Compumedics USA Inc., Charlotte, North Carolina, USA). During the recordings, the SEEG signal was referenced to a vertex screw/subdermal electrode and filtered between 0.05 and 500 Hz. The signal was sampled at 2000 Hz. A notch filter was applied at 50 Hz. All data were collected during the interictal stage. The stimulus-triggered electrical pulses were recorded along with the SEEG data for a precise synchronization with the stimulus onset.

### Electrode localization

The electrode placement was based solely on clinical requirements and was unaffected by this study’s needs. For each patient, we obtained a T1-weighted 1-mm isometric structural MRI scan using a 3-T Siemens scanner. After the implantation, a Siemens computed tomography (CT) scan was acquired. The reconstruction of the SEEG electrodes was performed using Brainstorm (Tadel et al. 2011), which is documented and freely available for download online under the GNU general public license (http://neuroimage.usc.edu/brainstorm). The post-implantation CT was co-registered to the preoperative anatomical MRI scan using the sequential pattern mining algorithms. Thus, the CT scan could be visualized on top of the preoperative MRI; however, there was a minimizing localization error due to a potential brain shift caused by surgery and implantation. Subsequently, recording sites were visually identified on the co-registered CT scan and marked in each subject’s preoperative MRI native space. The Montreal Neurological Institute (MNI) 152 structural template volume image was used to co-register with individual post-implantation CT scans to obtain the MNI coordinates, followed by a previously described protocol for the localization of the SEEG electrodes in the brain (Ashburner and Friston 2005; Fan et al. 2016). The definition of regions in the Human Brainnetome Atlas was shown by Fan et al. (Fan et al. 2016); it provides a 210 fine-grained cortical subregions. The BrainNet Viewer tool was applied to visualize the human brain subregions (Xia et al. 2013).

### Preprocessing and data analysis

For behavior analysis, the data were analyzed with GraphPad Prism (v.8.0; GraphPad Software). A paired t-test was performed to examine the mean reaction time (RT) in each of the two conditions for the Gaussian distribution of data. A Wilcoxon test was conducted to compare the mean accuracy in both conditions for the abnormal distributions of data. The data were presented as mean ± SEM and median ± quartile in case of the Gaussian and abnormal distributions, respectively. The Spearman correlation analysis was used between accuracies and ages, accuracies and gender, RT and ages, and RT and gender, respectively.

For SEEG analysis, the resting-state SEEG were evaluated by neurological and neurosurgical expertise, the sites recording containing ictal and inter-ictal activities would be excluded. The raw SEEG data were inspected visually to detect noisy/corrupted channels and exclude them from further analysis. Contacts within the white matter or cerebrospinal fluid were also excluded via co-registration of post-implanted CT and preoperative MRI images. The eligible data were all from performance with high accuracy (> 75%) during the cognitive tasks (for the work flow, see Supplemental Fig. 1). Finally, 85 electrodes with 348 recording sites in 20 patients were selected for further analysis (for detailed information, see Supplemental Table 1).

All SEEG data analyses were performed in MATLAB 2016b (Math Works Inc., Natick, MA) using Brainstorm (Tadel et al. 2011) and custom-developed analysis routines. Regarding the ERP analysis, the screened data were digitally filtered with a bandpass of 0.5–40 Hz. The epochs were selected from the 200-ms pre-S1 to 1000-ms post-S2 period, with overall 1700 ms for each one. The baseline was corrected by the average from the 200-ms pre-S1 to 0.5-ms pre-S1 interval. Sites containing over 30 trials in each condition would be used for further analysis.

For the time-frequency analyses, the 50 Hz power line interference (including its harmonics) was removed from the data. The spectrograms were calculated using Morlet’s wavelet transform with a linear step of 1 Hz over the range of 3–200 Hz. For each trial, we obtained its Morlet’s wavelet transform with the central frequency of 1 Hz and the time resolution (FWHM) of 3 s. The power bands were defined as the theta band (4–7 Hz), alpha band (8–13 Hz), beta band (14–29 Hz), gamma band (30–90 Hz) and high gamma band (91–200 Hz). For specific frequency band analysis, the Hilbert transform was applied to obtain the average power in each frequency section. Especially, for analyzing the high-gamma-band activities (also known as the high frequency oscillations, HFOs), after the specific band filtering (91–200 Hz), a threshold for HFOs was set three standard deviations above the mean baseline with at least three consecutive peaks.

The Permutation test was utilized to examine the mean SEEG amplitude or time-frequency power for each of the two conditions due to the abnormal distributions. To reduce the chances of obtaining a false-positive test, a false discovery rate (FDR) correction was used to adjust for multiple comparisons at P < 0.05. All statistical tests were two-sided unless stated otherwise. The p < 0.05 were considered statistically significant. Plotting was performed with R and GraphPad Prism (v.8.0; GraphPad Software).

## Results

### Behavior analysis and the ERPs recordings of numerical comparison and mental subtraction

**Figure 1:**
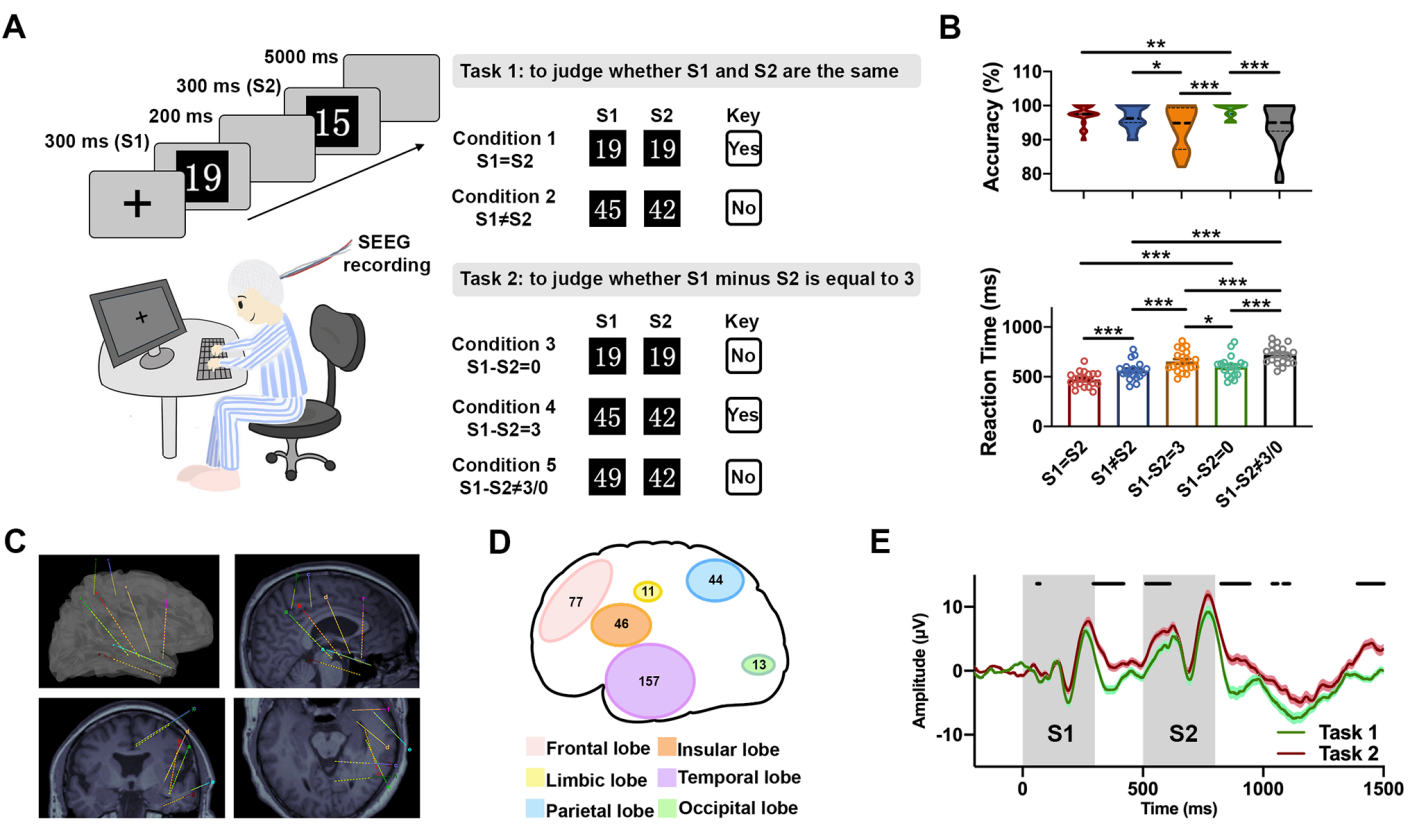
Experimental design and the SEEG recording in the human brain. **A,** the illustration of Tasks 1 and 2. The subjects confirmed the answer (“YES” or “NO”) for each task by pressing the right and left buttons of the mouse that had been balanced. In one trial, the paired digit (S1 > S2) that ranged from 11 to 49 were sequentially appeared for 300 ms respectively, with an interval of 200 ms. The interval between two trials was 5 s. Five conditions (“S1=S2”, “S1≠S2”, “S1-S2=0”, “S1-S2=3” and “S1-S2≠3/0”) were contained in two tasks. **B,** behavior analysis of five conditions in Task 1 and 2. Upper panel: the accuracies of the five conditions. The data were presented as median ± quartile. A Wilcoxon test was performed to examine the mean correct rate for each of the two conditions. Bottom panel: the reaction times of the five conditions. The data were presented as mean ± SEM. A paired t-test was conducted to assess the mean reaction time for each of the two conditions. * p<0/05, ** p<0.01 and *** p<0.001. **C,** the example reconstruction of the depth electrodes into the brain (Patient #15). The surface of the peripheral images (left-top) show the reconstruction of eight electrodes into the brain of Patients #15. The lateral view (right-top), the coronal view (left-bottom) and the top views (right-bottom) of the reconstructed electrode were based on the three-dimensional co-registered MRI. **D,** the distribution counts of the 348 SEEG recording sites in the cortex lobes. **E,** the average ERPs amplitudes of total 348 electrodes of Task 1 (green line) and 2 (red line), with the shadow as the SEM, respectively. The grey rectangle indicated the durations of S1 and S2. The black bar presented the t-test between Tasks 1 and 2 with p<0.01.

The subjects were instructed to complete two tasks, consisting of two runs each. They were presented with a pair of sequenced digital numbers (S1 > S2) and required to assess whether S2 was identical to S1 in Task 1. Subsequently, the participants were requested to examine whether the difference between S1 and S2 (S1-S2) was equal to 3 in Task 2 (Fig. 1a). Overall, the following five conditions were evaluated: “S1 = S2”, “S1 ≠ S2”, “S1-S2 = 0”, “S1-S2 = 3” and “S1-S2 ≠ 3/0”. The statistics for the behavioral data showed that the accuracy in the five conditions were 97.50% (97.50, 100), 96.25% (95.00, 100), 100.00% (97.56, 100.00), 94.87% (87.18, 99.36) and 95.00% (92.50, 100), respectively (Wilcoxon matched-pairs signed rank test; Fig. 1b-upper panel). The RTs, which were the intervals between the onset of S2 and the time when the answer key was pressed, were 472.1 ± 16.99 ms, 561.30 ± 20.53 ms, 600.10 ± 23.10 ms, 654.10 ± 23.65 ms and 720.50 ± 20.73 ms, respectively (paired t-test; Fig. 1b-bottom panel). All participants performed the two tasks with a high accuracy, and their RT patterns reflected the most widely replicated behavioral effect in the numeric cognitive arithmetic: the problem size effect (Ashcraft 1992). Specifically, the behavioral data indicated the degree of difficulty across the conditions with an order of “S1 = S2”< “S1 ≠ S2”, “S1-S2 = 0”< “S1-S2 = 3”< “S1-S2 ≠ 3/0”. The correlation analysis suggested no significant correlations between the participants’ sex or age and the behavioral data, respectively (Supplemental Table 2).

The implanted electrodes were reconstructed through the co-registered preoperative MRI and the postimplantation CT (Fig. 1c). Out of the total 348 recording sites, 157 (45.11%), 77 (22.13%), 46 (13.22%), and 44 sites (12.64%) were located in the temporal, frontal, insular, and parietal lobes, respectively. In addition, 13 sites (3.74%) were within the occipital lobe, and 11 sites (3.16%) in the limbic lobe (Fig. 1d; for detailed information of the subregional distribution, see Supplemental Table 3). The SEEG was segmentally extracted to ERP epochs between the 200-ms pre-S1 and 700-ms post-S2 period (total 1700 ms). The average epochs in five conditions were rearranged based on the lobar distribution of the sites (Supplemental Fig. 2A). The amplitudes of the average epochs of the ERPs showed significant differences during the cognition activities in Tasks 1, Task 2 (Supplemental Fig. 2B-C), and between Task 1 and 2, respectively (Fig. 1e). It is suggested the human brain goes through a discriminative cognition processing during simple numeric comparison and mental subtraction. However, the precise pathway of this distinguished cognition controls should be explored.

### Numerical and subtraction results comparison set the time window of digital subtraction

The procedure of mental subtraction was initiated immediately after the onset of S2. However, during the paradigms, the introduction of the task must have affected the participants’ attention and motivation. This attentional effect would be an interference of the procedure of mental subtraction. Especially in the Task 2, two types of incongruency processing were mixed, including the incongruency processing of visual acquired numbers as the precondition for subtraction (Prado et al. 2011; Gómez-Velázquez et al. 2015), and the incongruency processing of discriminating the results from the enquired ones 3 after mental subtraction.

**Figure 2:**
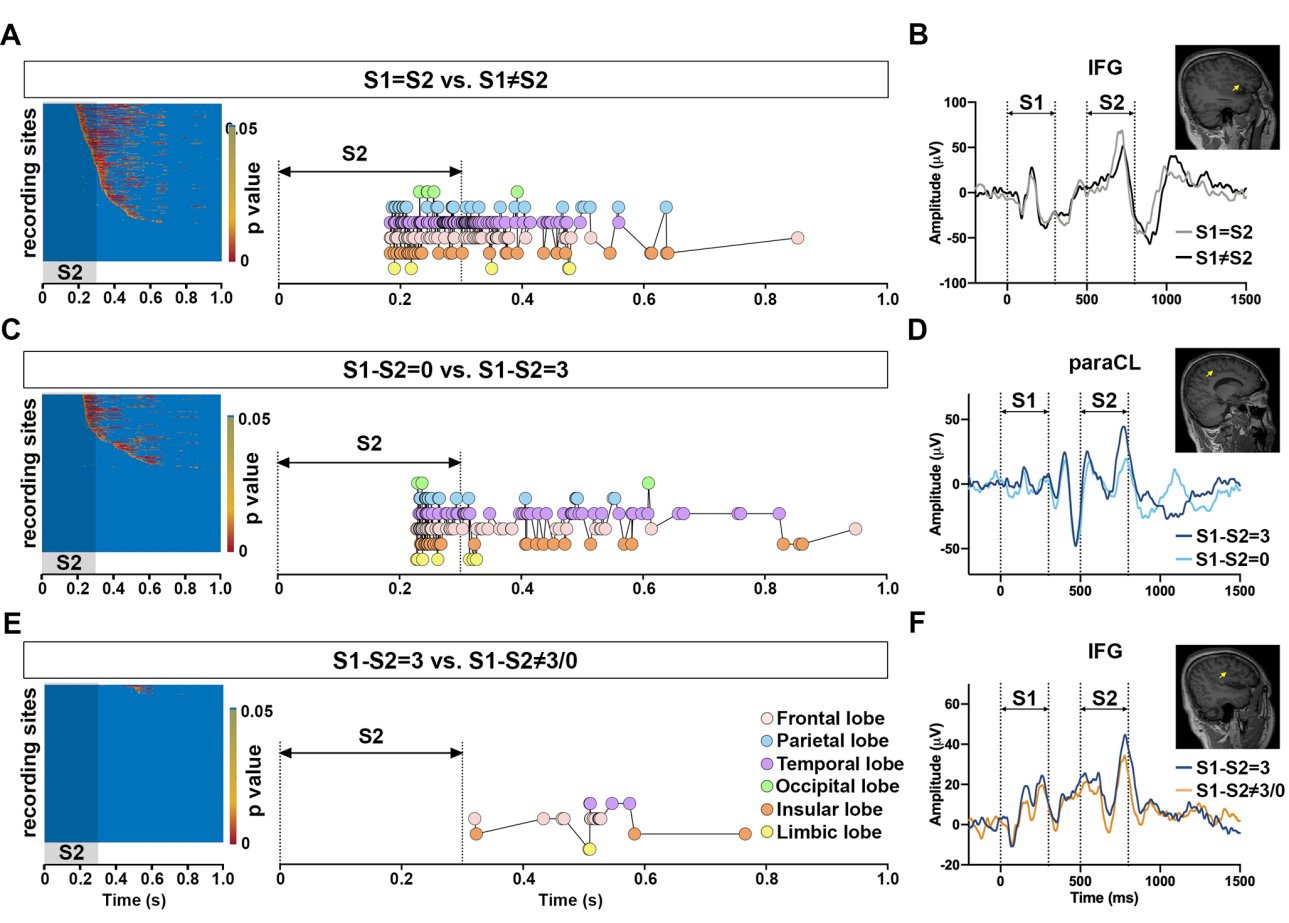
The incongruent processing of the numerical and subtraction comparison. **A,** left panel: the heatmap of the p-value from the nonparametric test of the SEEG amplitude of the recording sites examined in conditions 1 vs. 2 (S1=S2 vs. S1≠S2) after the S2 onset. Each row represented one recording site. The p-values were ordered as the occurrence time when the FDR corrected p<0.05 for individual recording sites. The values of p>0.05 were shown as the background (blue). The shadow rectangle indicated the duration of S2. Right panel: the time lapse of each recording site from the heatmap on the left panel with p<0.05. The different colored bars represented cortical lobes, in which the recording sites were distributed: pink, frontal lobes; blue, parietal lobes; purple, temporal lobes; green, occipital lobes; orange, insular lobes and yellow, limbic lobes. The vertical dotted lines indicated the duration of S2. **B,** the average trace of the ERPs of the earliest recoding site with p<0.05 in (A), under the conditions of S1=S2 (grey line) and S1≠S2 (black line). The site, located at the IFG, was indicated as the yellow arrow in the sagittal MRI individually (insertion). **C,** left panel: the heatmap of the p-value from the nonparametric test of the SEEG amplitude of the recording sites examined in conditions 3 vs. 4 (S1-S2=0 vs. S1-S2=3) after the S2 onset. Each row represented one recording site. The p-values were ordered as the occurrence time when the FDR corrected p<0.05 for individual recording sites. The values of p>0.05 were shown as the background (blue). The shadow rectangle indicated the duration of S2. Right panel: the time lapse of each recording site from the heatmap on the left panel, with p<0.05. The different colored bars represented the cortical lobes, in which the recording sites were distributed: pink, frontal lobes; blue, parietal lobes; purple, temporal lobes; green, occipital lobes; orange, insular lobes and yellow, limbic lobes. The vertical dotted lines indicated the duration of S2. **D,** the average trace of the ERPs of the earliest recoding site with p<0.05 in (C), under the conditions of S1-S2=0 (light blue line) and S1-S2=3 (dark blue line). The site, situated at the paraCL, was indicated as the yellow arrow in the sagittal MRI individually (insertion). **E,** left panel: the heatmap of the p-value from the nonparametric test of the SEEG amplitude of the recording sites examined in condition 4 vs. 5 (S1-S2=3 vs. S1-S2≠3/0) after the S2 onset. Each row represented one recording site. The p-values were ordered as the occurrence time when the FDR corrected p<0.05 for the individual recording sites. The values of p>0.05 were displayed as the background (blue). The shadow rectangle indicated the duration of S2. Right panel: the time lapse of each recording sites from the heatmap on the left panel, with p<0.05. The different colored bars represented cortical lobes, in which the recording sites were distributed: pink, frontal lobes; blue, parietal lobes; purple, temporal lobes; green, occipital lobes; orange, insular lobes and yellow, limbic lobes. The vertical dotted lines indicated the duration of S2. **F,** the average trace of the ERPs of the earliest recoding site with p<0.05 in (E), under the conditions of S1-S2=3 (blue line) and S1-S2≠3/0 (orange line). The site, located at the IFG, was indicated as the yellow arrow in the sagittal MRI individually (insertion).

To extract the core process of mental subtraction, we attempted to set a time frame for the subtraction in the human brain. Thus, a time-lapse reordering was applied to the heatmap of the FDR-corrected p-values, which resulted from the comparison of the SEEG amplitude of each recording site in conditions 1 vs. 2 (“S1 = S2” vs. “S1 ≠ S2”, Fig. 2a), 3 vs. 4 (“S1-S2 = 0” vs. “S1-S2 = 3”, Fig. 2c), and 4 vs. 5 (“S1-S2 = 3” vs. “S1-S2 ≠ 3/0”, Fig. 2e). The comparison represented the differences between two cognitive control activities, which were numeric comparison and digital subtraction with the same introduction, respectively. In “S1 = S2” vs. “S1 ≠ S2”, the comparison only presented the incongruency processing of simple digitals. Since the SEEG amplitudes of the IFG site demonstrated the earliest significant difference at 183.5 ms after the S2 onset (Fig. 2b), the result indicated the initiation timepoint of numeric comparison (183.5 ms after S2 onset, Fig, 2a) and subregion in the brain (IFG, Fig. 2b). In “S1-S2 = 0” vs. “S1-S2 = 3”, the comparison contained the incongruency processing of both digital and subtraction results. Notably, the peak amplitudes of the paracentral lobule (paraCL) site indicated the earliest difference emerged at 228 ms after the S2 onset (Fig. 2d). In “S1-S2 = 3” vs. “S1-S2 ≠ 3/0”, the comparison only presented the incongruency processing of the subtraction results, that is the result-decision after the subtraction operation in the brain. Furthermore, the peak amplitudes of the IFG site primarily showed the earliest difference at 320.5 ms after the S2 onset (Fig. 2f). The result indicated the termination timepoint of subtraction operation (320.5 ms after S2 onset, Fig, 2e) and subregion in the brain (IFG, Fig. 2f). Therefore, the result indicated that the core process of mental subtraction would proceed between the numeric comparison and subtraction results comparison, which was between 183.5 ms and 320.5 ms from the S2 onset.

### Temporal-spatial comparison of numerical comparison and subtraction

**Figure 3:**
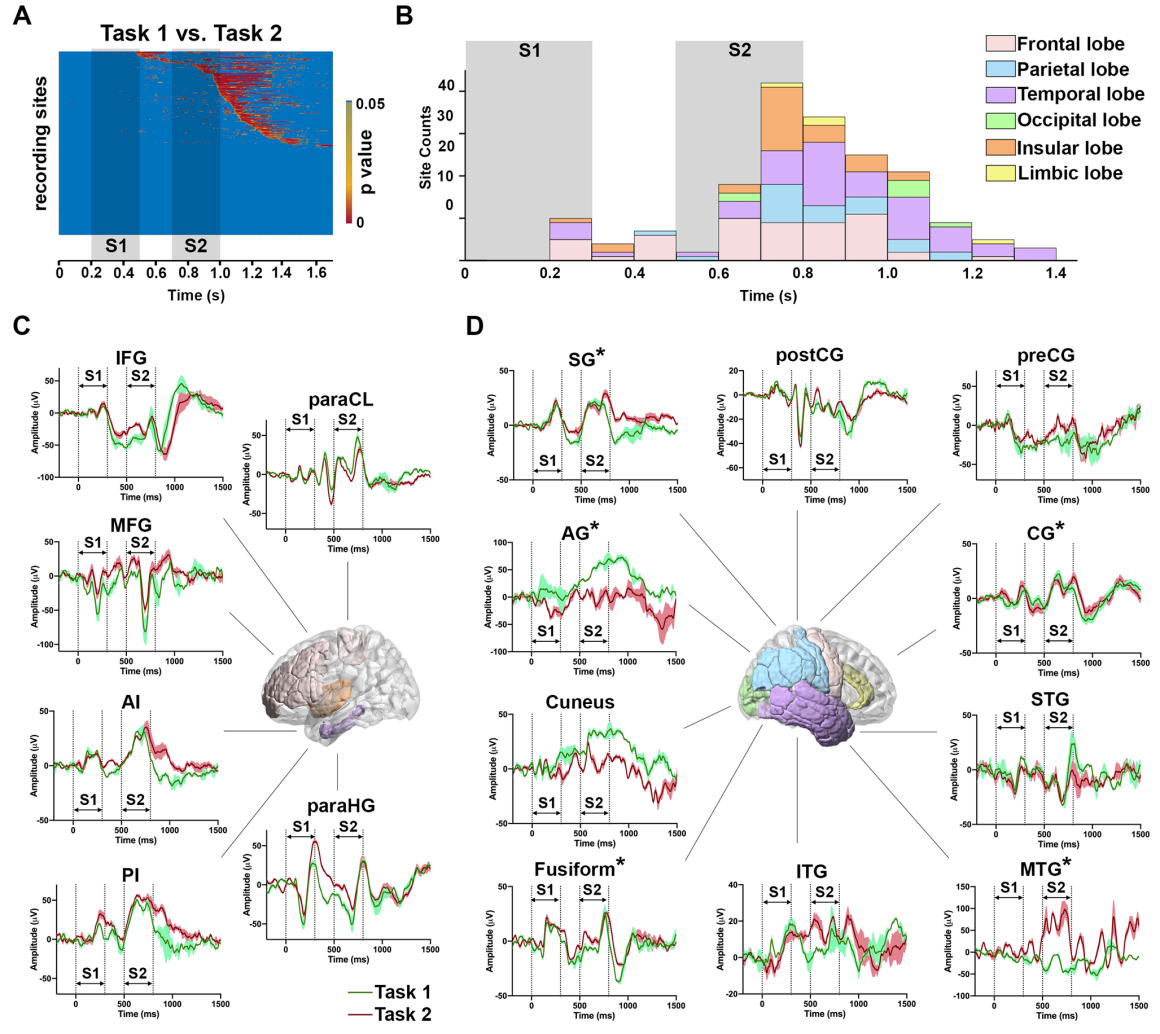
The comparison of numerical comparison and subtraction. **A,** the heatmap of p-value from the nonparametric test of the SEEG amplitude of 348 recording sites examined in Tasks 1 (numerical comparison) vs. 2 (digital subtraction). Each row represented one recording site. The shadow rectangles indicated the durations of S1 and S2, respectively. The p values were ordered as the occurrence time when the FDR corrected p<0.05 (continuous for 50 ms) for individual recording sites. The values with p>0.05 were shown as the background (blue). **B,** the distribution counts of the recording sites at different time periods after the S1 onset in (A). The interval time was 100 ms. The shadow rectangles signified the duration of S1 and S2, respectively. The different colored bars represented cortical lobes, in which the recording sites were distributed: pink, frontal lobes; blue, parietal lobes; purple, temporal lobes; green, occipital lobes; orange, insular lobes; and yellow, limbic lobes. **C,** the example the ERPs traces of the recording sites distributed in the paraCL, IFG, MFG, AI, PI, and paraHG. The ERPs amplitudes were significantly different between Tasks 1 (green line) and 2 (red line) during S1 and S2 (p<0.05 continuous for 50 ms), with the shadow as the SEM, respectively. The location of each region was shown in the brain template: pink, frontal lobes; purple, temporal lobes; and orange, insular lobes. The vertical dotted lines indicated the duration of S1 and S2. **D,** the example the ERPs traces of the recording sites distributed in the preCG, postCG, SG, AG, cuneus gyrus, fusiform gyrus, ITG, MTG, STG and CG. The ERPs amplitudes were significantly different between Tasks 1 (green line as the average and shadow as the SEM) and 2 (red line as the average and shadow as the SEM) only during S2 (p<0.05 continuous for 50 ms), while there was no significant change during S1. The location of each region has been shown in the brain template: pink, frontal lobes; blue, parietal lobes; purple, temporal lobes; and yellow, limbic lobes. The recording sites, with the ERPs amplitudes were significantly different between Tasks 1 and 2 after 182 ms of the S2 onset, were indicated with a star. The vertical dotted lines indicated the durations of S1 and S2.

To explore the temporal-spatial progressing of mental subtraction, the ERPs amplitudes of each recording site were compared between Tasks 1 and 2. The FDR-corrected p-value (p < 0.05 lasting over 50 ms) was also reordered in a time lapse (Fig. 3a). The chronological sites distributed in the brain lobes, within which the frontal, temporal, and insular lobes emerged distinct amplitudes of the ERPs between numeric comparison and subtraction early after 200 ms of the S1 onset (Fig. 3b). Furthermore, the majority of the sites had reacted at the differences between tasks since 100 ms after the S2 onset.

Among these aforementioned sites, those distributed in the paraCL, IFG and MFG of the frontal lobes, the anterior insula (AI) and posterior insula (PI) lobes, and the temporal parahippocampous (paraHG), demonstrated the different amplitudes of the ERPs between two tasks before S2 and during S2 (Fig. 3c). This might indicate that more than subtraction, these regions would be involved in other cognitive activities. However, the sites distributed in the frontal preCG, the cingulate gyrus (CG) in the limbic lobe, the postCG, SG, and AG of the parietal lobes, the occipital cuneus gyrus, the fusiform gyrus, the inferior temporal gyrus (ITG), MTG, and STG of the temporal lobes were presented differences between Tasks 1 and 2 during S2 (Fig. 3d). The sites in the parietal lobes emerged with a large proportion during S2 than before it. These results suggested that the parietal region could play a critical role in mental subtraction in the human brain. Further, diverse brain regions participated in the consecutive procedures of cognitive control, respectively. Especially, recordings from five sites in the SG, one site in the AG, two sites in the fusiform, four sites in the MTG, and two sites in the CG within the parietal-cingulate-temporal cortices initiated the differences after 183 ms of the S2 onset that might be considered as an origin of subtraction based on the aforementioned results. Therefore, the mechanism of discriminating numeric comparison and subtraction in the core regions, including the SG, AG, fusiform, MTG, and CG, ought to be clear.

### Gamma band activities undertake digital subtraction chronologically

**Figure 4:**
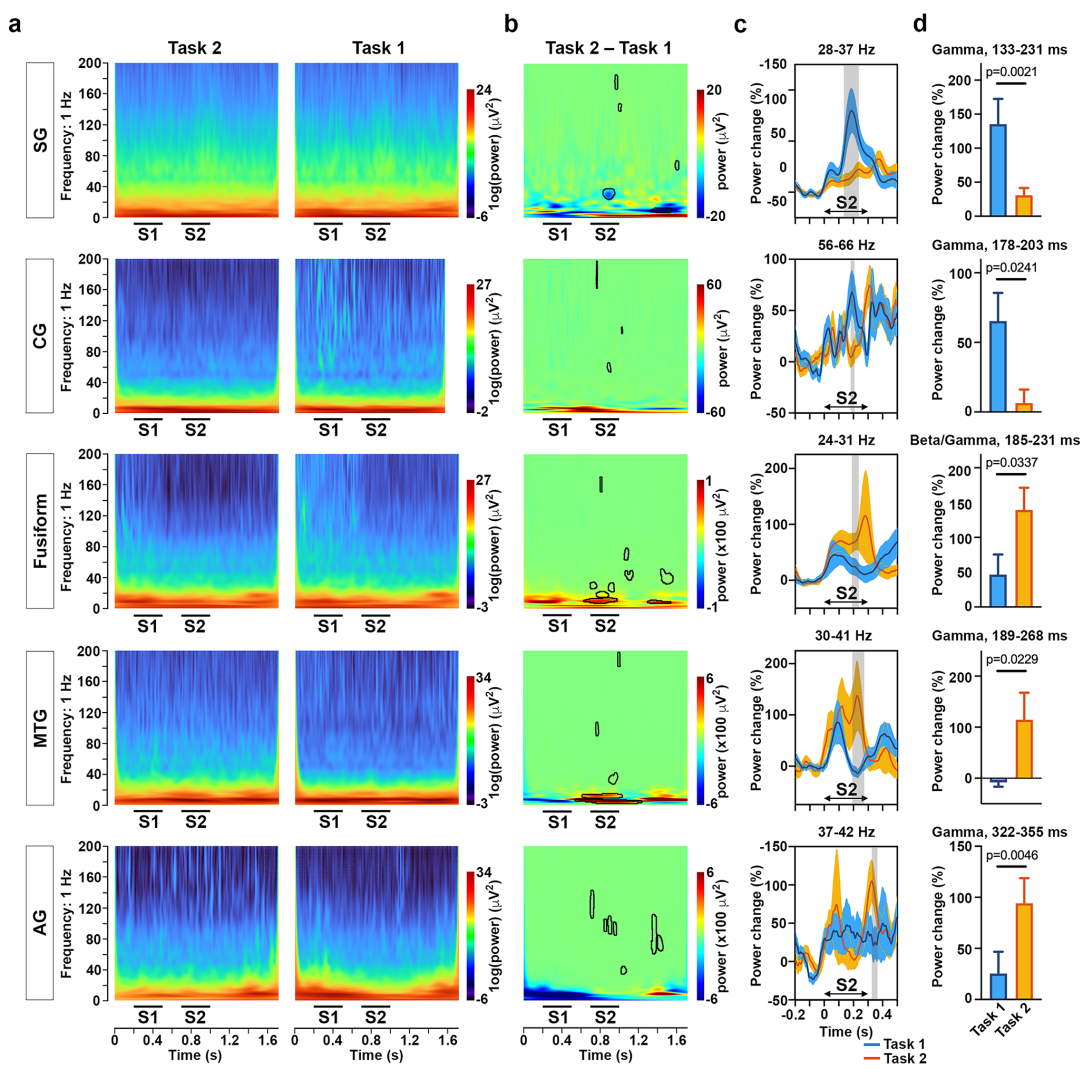
Time-frequency analysis for numerical comparison and subtraction. **A,** time-frequency representations of the power response relative to Tasks 2 and 1 of the earliest recording sites, with discriminable ERPs amplitudes of Task 2 from 1, distributed at the SG, CG, fusiform gyrus, MTG, and AG, respectively. The black lines underneath the heatmap indicated durations of S1 and S2, respectively. **B,** time-frequency representation of the power response difference between Tasks 2 and 1 of the five regions in (A), showing significant decreased (blue) or increased (red) activity. Significant clusters (FDR corrected p<0.05, non-parametric permutation test) have been encircled with a solid black line. **C,** normalized power of the activities at the identified frequency band (gamma or beta/gamma) of five regions in (A) over time, respectively. Significant differences between Tasks 1 (blue line as the mean and shadow as the SEM) and 2 (orange line as the mean and shadow as the SEM) have been marked by a shadowed bar (p<0.05, t-test). The arrowed line indicated the duration of S2. **D,** The average spectral power relative to the baseline activity in the identified time period and frequency band for Tasks 1 (blue) and 2 (orange) in (C) (p<0.05, t-test).

Brain oscillations at different frequency bands, even the high-gamma-band, have been shown to play a key role in various cognitive tasks, including memory, executive control, and attention to internal processing or the external environment (Klimesch 1999; Kawasaki et al. 2010; Gaona et al. 2011; Kucewicz et al. 2014; Akiyama et al. 2017). Therefore, further exploration of the local network in the frequency domain would provide relevant information regarding the neural mechanism of mental subtraction. The power spectra normalized to each frequency in the five core regions, including the SG, AG, fusiform, MTG and CG, spanning across 3–200 Hz were compared between numeric comparison (Task 1) and subtraction (Task 2) (Fig. 4a). The permutation tests were applied to the power spectra in response to subtraction and numeric comparison. This identified clusters with significant event-related differences once after the S1 onset for the two tasks (p.cluster < 0.05, Fig. 4b). The sparse clusters in the high gamma range (> 90 Hz) during S2 were found in all of the five core regions. However, the analysis of high gamma activities (known as the high frequency oscillations, HFOs) showed that there were sparse HFOs during recordings of Task 1 and 2, respectively. And there was no significant difference of the HFOs rate between two tasks (Supplemental Fig. 3). Notably in fusiform, the alpha band activities with a long latency (9–14 Hz, 68 ms before S2 to 263 ms after S2) and early emerging beta band activities (17–22 Hz, 60–177 ms of S2 ) demonstrated higher powers in Task 2 than in Task 1. Moreover, in the MTG, there were consistent theta and alpha band activities (for theta, 4–8 Hz, 130 ms before S2 to 462 ms after S2; for alpha, 12–15 Hz, 37 ms before S2 to 320 ms after S2), with a higher power in Task 2 than that in Task 1. The low frequency activities in the two temporal lobes suggested a background of mental subtraction, such as calculating attention and focusing during the two different tasks.

Besides the high gamma and low theta/alpha band, the clusters in gamma or beta/gamma ranges in the five regions presented a chronological order (Fig. 4c): in the SG, the gamma activities in the range of 28–37 Hz at a short latency (133–231 ms of S2 onset) indicated a higher power in Task 1 than in Task 2; in the CG, the gamma activities in the range of 56–66 Hz at a short latency (178–203 ms of S2 onset) showed a greater power in Task 1 than that in Task 2; in the fusiform gyrus, the beta/gamma activities in the range of 24–31 Hz at a latency of 185–231 ms demonstrated a higher power in Task 2 than that in Task 1; in the MTG, the gamma activities in the range of 30–41 Hz at a latency of 189–268 ms showed a greater power in Task 2 than that in Task 1; and in the AG, the gamma activities in the range of 37–42 Hz at a latency of 322–355 ms showed a greater power in Task 2 than that in Task 1 (Fig. 4d).

The power of the activities at the identified frequency band produced differences between numeric comparison and subtraction sequentially. This raised the question as to whether those clusters at a short latency reflects a modulation of oscillations. To address this query, we examined the ERPs latency of the sites, the amplitude of which showed differences during 183–322 ms of the S2 onset. The latency of the sites indicated differences in the following order (early to late): the MTG, SG, CG, fusiform, and AG (Fig. 3d). These analyses demonstrated that the gamma or beta/gamma power in the five regions might not be driven by the phase-locked ERP activities. Therefore, we referred to those chronological frequency power changes between Tasks 1 and 2 as activities rather than oscillations.

**Figure 5:**
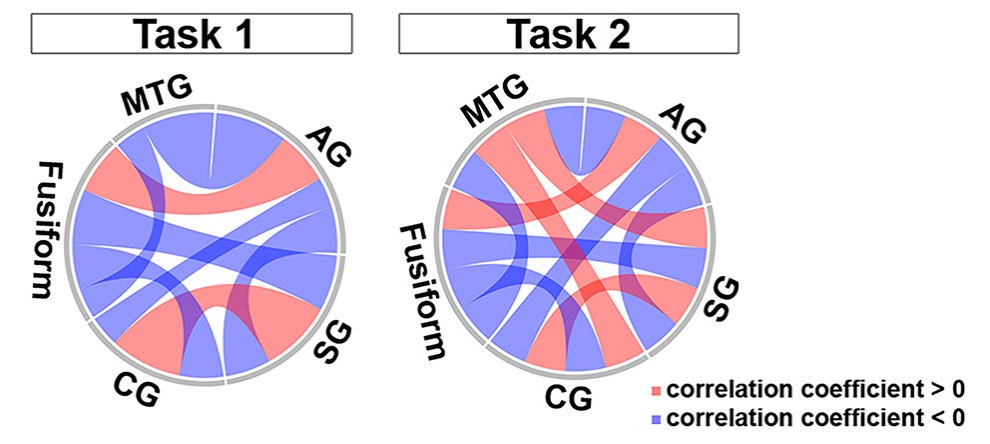
Correlation of five core regions in numerical comparison and subtraction.Chord diagrams of correlations among the typical gamma band power of the core regions, including SG, CG, fusiform, MTG, and AG, in Task 1 and 2, respectively. The Spearman correlation were applied in each two regions among SG (power of 28?37 Hz, during 133?163 ms), CG (power of 56?66 Hz, during 178?208 ms), fusiform (power of 24?31 Hz, during 185?315ms), MTG (power of 30?41 Hz, during 189?219 ms), and AG (power of 37?42 Hz, during 322?352 ms), respectively. The average correlation coefficient during the initiated 30 ms of five regions were plotted, with the red and blue link indicated the positive and negative correlation, respectively.

To verified the intrinsic effects among aforementioned five regions, a Spearman correlation was conducted in the power at the typical frequency band and time duration. The correlation networks indicated that effective correlations among five regions existed in Task 1 and 2, however, there were not effective connection between the MTG and CG, and the MTG and fusiform in Task 1, while as the connections existed in Task 2 (Fig. 5). Notably, most of correlations, such as the AG-CG, the AG-fusiform, the AG-MTG, the SG-CG, the SG-fusiform, the SG-MTG, and the CG-MTG in Task 1 were opposite to those in Task 2. Exceptionally, the correlation of the AG-SG, the CG-fusiform, and the fusiform-MTG showed the consistently negative correlation in Task 1 and 2. The results provided a possibility that the gamma band activities in the SG, CG, fusiform, MTG, and AG might follow a causal relationship to mediated subtraction process, with a distinguished network connections from that in simple numeric comparison.

## Discussion

Our findings, based on the most extensive and precise description of the local field potentials in mainly the human neocortical regions, revealed several important insights about the neural mechanisms underlying mental subtraction in the human brain. Mental subtraction requires a complex interplay between many brain regions. Omitting the visual perceptual information (early S1 duration), the initiation times of numeric comparison and subtraction results comparison, set a time frame for the core mental subtraction process, which was from 182 ms to 322 ms after the S2 onset. Our results provided evidence that before showing the S2, the arithmetic-dependent attention was a hybrid procedure in the anterior regions, including paraCL, IFG, MFG, AI, PI, and paraHG. Furthermore, after the S2 onset, the middle-to-posterior regions were activated to participate into mental subtraction, including the preCG, postCG, SG, AG, cuneus and fusiform gyri, ITG, MTG, STG, and CG. Among these regions, the SG, CG, fusiform, MTG, and AG demonstrated significant differences between two tasks within the time frame of mental subtraction. Moreover, the gamma or beta/gamma band activities in the five regions were chronologically involved in the mental subtraction process. At this point, the temporal-spatial mechanism of mental subtraction in the human brain was deciphered for the first time.

Converging evidence has identified the frontoparietal network as the main cortical frame that supports mental arithmetic. Using the fMRI, regions including the IFG, insular, paraCL, left fusiform gyrus, visual area, superior and inferior parietal lobules, and CG were common to both number and calculation tasks; additionally, the AG, MFG, and SFG showed activity in calculation tasks (Arsalidou and Taylor 2011). However, the examining technique cannot offer a high temporal resolution during tasks. Our results predicted that the frontal regions would be participate since the early stage of mental subtraction (before S2), while the parietal regions would be dominantly active during the subtraction proceeding (during S2). The time frame set by different conditions within the same tasks could restrict the course of mental subtraction from an interference with the external instruction-driving motivation and intrinsic attention.

In our study, the ERPs in both IFG and MFG presented different peak amplitudes for numbers than that for subtraction before the calculations. This result could be an verification regarding the frontal lobes underling the strategy choice and planning in mathematical processes (Dehaene and Cohen 1997). It has been proposed that the posterior-anterior progression in the insula could play a hub role before mental subtraction (Chang et al. 2013). The distinguishing of the PI, following the AI, in the ERPs reaction to Tasks 1 and 2 suggested that they were responsible for switching the brain networks during the information processing. As the hippocampus’ neighboring structure, the paraHG is thought to be associated with information recollection to retrieve the subtraction fact rather than the previous numbers (Bloechle et al. 2016). The paraCL, which is often referred to as the SMA, performed at a higher peak amplitude near before the S2 onset in subtraction than that in numbers. Studies provided evidence for the sharing regions, including the SMA, regarding the arithmetic and finger representation (Michaux et al. 2013; Berteletti and Booth 2015). Considering the SMA’s crucial role in representations for finger movements (Diedrichsen et al. 2013), the corresponding findings indicated the possibility that it underlies finger perception when subjects engage in subtraction problem-solving. Thus, before the core subtraction ongoing in the human brain, several regions cooperate to solve a mathematical problem, including choosing the strategy, preparing for fact retrieval, and networks switching.

Entering the S2 duration was followed by the goal-oriented proceeding of the numeric comparison and mental subtraction tasks. In addition to those regions mentioned above, more regions, especially the parietal and temporal regions were typically involved in this procedure. According to the order of latency of the sites that emerged difference between Tasks 1 and 2 (early to late), the caudoventral ITG showed a higher peak amplitude of subtraction than numbers as soon as S2 was revealed. However, recent studies using an intracranial EEG have found the posterior ITG to be activated during the visual perception of numbers and spreading its adjacent connection in calculations (Pinheiro-Chagas et al. 2018), which might be later than the time of the S2 onset. Therefore, the ITG would play a more critical role in calculation than in numbers. Considering the SMA’s role prior to the S2 onset, the ITG and STG were discovered as reflecting attentional orienting toward the fingers when performing arithmetic problems (Proverbio and Carminati 2019). The other selected regions with different reactions between numbers and subtraction, including the postCG, preCG, SG, AG, occipital cuneus gyrus, fusiform, CG, and MTG were reported to participate in the calculation in the human brain (Ischebeck et al. 2006, 2009; Abd Hamid et al. 2011; Arsalidou and Taylor 2011; Ni et al. 2011; Liu et al. 2017). Among the regions that began to discriminate numbers and subtraction, it was quite expected to observed the mechanism of cooperation of the core regions during the time window of 182 ms to 322 ms of the S2, when it was supposed to proceed the critical mental subtraction.

Several results from the frequency analysis revealed that various band activities were involved in calculation. Most studies focused on the theta and alpha band activities in the frontoparietal regions in subtraction (De Smedt et al. 2009a; Kitaura et al. 2017). However, the involvement of the beta and gamma bands in arithmetic remains insufficiently known. In mixed arithmetic problem solving, both the alpha and beta band powers in the frontal-parietal network were suppressed as a function of attention load (Lin et al. 2015). Furthermore, in the serial subtraction task, the gamma ERS increased in the right IPS and the gamma ERD decreased in the IFG using the MEG techniques (Ishii et al. 2014). Our findings provided evidence that as the time progressed during the critical time window of mental subtraction, the power of gamma band activities temporally and spatially changed in mental subtraction, compared with the basic numeric processing, along with the changes of theta and alpha band activities in the temporal lobes. It emphasized the important role of the gamma band activity in mental subtraction, especially in the parietal and temporal cortices. This neural basis of the gamma-mediated information flow in the posterior cortex might be able to provide novel insights on the higher cognitive function besides subtraction.

There are, of course, several limitations that should be mentioned. First, all participants were implanted with unilateral depth electrodes, and we could not resolve the lateralization of these responses or assess whether these effects were larger in the left or the right hemisphere. Second, we attempted to extract the cognitive activity related to mental subtraction by comparing it with that associated with the numeric incongruity processing. The two tasks could undergo the same visual perception of the digital Arabic numbers, however, with different instructional attention and memory. Therefore, we failed to provide a direct evidence for precise initiation of subtraction operation. Third, to decrease the problem size, the participants were required to respond by pressing the key to indicate the subtraction solution was the same as number 3. In regular mental subtraction, participants would judge whether the calculation result equal to the random correct answer. Future research would need to generally examine how the relationship between intact number processing and mental arithmetic is modulated by strategy on a trial-by-trial basis. Last, we would like to acknowledge that our exploration was data driven. Other attempts of neural modulation, such as the cortico-cortical evoked potentials originating from SEEG, could be helpful for verifying the mechanism of dynamic mental subtraction in the human brain. Hence, further investigation may provide stronger and additionally detailed evidence of this relationship.

In summary, using the SEEG recording under the numeric subtraction tasks, our results confirmed and extended the previous studies findings indicating that mental subtraction were taken in a critical time window in the human brain. Moreover, this study’s findings suggested that before the window, the anterior cortex, including the paraCL, IFG, MFG, AI, PI, and paraHG were dominantly involved. During the window, the gamma band activities would act as a crossover in the posterior regions, including the SG, CG, fusiform, MTG, and AG within the parietal-cingulate-temporal cortices, to proceed the core procedure of mental subtraction. Our results complement previous work regarding numeric comparison and mental subtraction by providing deeper insights into the neural basis of the mental arithmetic. The temporal-spatial mechanism underlying mental arithmetic might provide essential insights with respect to the higher cognition neuroscience.

### Electronic supplementary material

Below is the link to the electronic supplementary material.

Supplementary Material 1

## Data Availability

The original data used in this study included SEEG data, pre-implanted MRI and post-implanted CT images for 20 patients with pharmacologically resistant epilepsy at the Xuanwu Hospital, Capital Medical University, Beijing, China. Any original data request to the authors should be applied and permitted by hospital administration.

## Abbreviations

AG: Angular gyrus
AI: Anterior insula
CG: Cingulate gyrus
DMS: Delayed match/mismatch-to sample
ERD: Event-related desynchronization
ERP: Event related potential
ERS: Event-related synchronization
IFG: Inferior frontal gyrus
ITG: Inferior temporal gyrus
MFG: Middle frontal gyrus
MTG: Inferior temporal gyrus
paraCL: Paracentral lobule
paraHG: Parahippocampous
PI: Posterior insula
postCG: Postcentral gyrus
preCG: Precentral gyrus
RT: Reaction time
SG: Supramarginal gyrus
SMA: Supplementary motor area
STG: Superior temporal gyrus
