# Supplementary material for: Temporal-spatial deciphering mental subtraction in the human brain
Source: Cogn Neurodyn. 2023 Feb 27;18(3):893–906. doi: 10.1007/s11571-023-09937-z (PMC11143099; doi:10.1007/s11571-023-09937-z)
Supplement: Supplementary file 1 — Supplementary Material 1 [file 11571_2023_9937_MOESM1_ESM.docx]

## **Supplemental Table 1: Profiles of 20 patients.**

| Participant NO. | Gender (F/M) | Age (years) | Available sites count | Available electrodes count |
| --- | --- | --- | --- | --- |
| 1# | M | 26 | 4 | 3 |
| 2# | M | 23 | 14 | 4 |
| 3# | F | 27 | 10 | 5 |
| 4# | M | 19 | 23 | 4 |
| 5# | F | 30 | 8 | 4 |
| 6# | M | 23 | 39 | 7 |
| 7# | M | 22 | 46 | 8 |
| 8# | M | 18 | 32 | 6 |
| 9# | M | 26 | 10 | 2 |
| 10# | F | 13 | 3 | 1 |
| 11# | M | 22 | 13 | 4 |
| 12# | M | 27 | 23 | 5 |
| 13# | M | 24 | 5 | 3 |
| 14# | F | 16 | 1 | 1 |
| 15# | M | 41 | 29 | 7 |
| 16# | M | 35 | 13 | 3 |
| 17# | F | 52 | 11 | 2 |
| 18# | M | 28 | 26 | 5 |
| 19# | M | 24 | 23 | 5 |
| 20# | M | 17 | 15 | 6 |

## **Supplemental Table 2: Correlation analyses of accuracies and RTs, with participants’ gender or age.**

|  |  |  | Accuracies | | RT | |
| --- | --- | --- | --- | --- | --- | --- |
|  |  |  | coefficient | P-value | coefficient | P-value |
| Task 1 | Correlating to gender | S1=S2 | 0.2833 | 0.2134 | 0.02418 | 0.9172 |
|  |  | S1≠S2 | 0.09231 | 0.6907 | -0.2102 | 0.3604 |
|  | Correlating to age | S1=S2 | 0.04175 | 0.8574 | -0.00089 | 0.9969 |
|  |  | S1≠S2 | -0.02366 | 0.9189 | -0.06585 | 0.7767 |
| Task 2 | Correlating to gender | S1-S2=3 | -0.04401 | 0.8498 | 0.1524 | 0.5096 |
|  |  | S1-S2=0 | -0.15590 | 0.4997 | 0.3099 | 0.1716 |
|  |  | S1-S2≠3/0 | -0.1766 | 0.4439 | 0.1766 | 0.4438 |
|  | Correlating to age | S1-S2=3 | 0.08671 | 0.7086 | -0.2444 | 0.2857 |
|  |  | S1-S2=0 | 0.1938 | 0.3998 | 0.00456 | 0.9843 |
|  |  | S1-S2≠3/0 | -0.1116 | 0.6300 | -0.1368 | 0.5543 |

## **Supplemental Table 3: Subregions of individual cortical lobes locating the recording sites.**

| Lobes | Subregions | Site counts |
| --- | --- | --- |
| Frontal lobe |  | **77** |
|  | Middle Frontal Gyrus  Inferior Frontal Gyrus  Precentral Gyrus  Paracentral Gyrus  Orbital Gyrus  Superior Frontal Gyrus | 24  21  21  9  1  1 |
| Parietal lobe |  | **46** |
|  | Postcentral Gyrus  Supramarginal Gyrus  Angular Gyrus | 19  14  11 |
| Temporal Lobe |  | **157** |
|  | Superior Temporal Gyrus  Middle Temporal Gyrus  Inferior Temporal Gyrus  Parahippocampal Gyrus  Posterior Superior Temporal Sulcus  Fusiform Gyrus | 39  37  36  24  12  9 |
| Insular lobe |  | **46** |
|  | Posterior insula  Anterior insula | 29  17 |
| Limbic lobe |  | **11** |
|  | Cingulate Gyrus | 11 |
| Occipital lobe |  | **13** |
|  | Occipital Gyrus  Cuneus  Superior Occipital Gyrus | 7  5  1 |

**Supplemental Fig. 1 Experimental flow chart.**

**
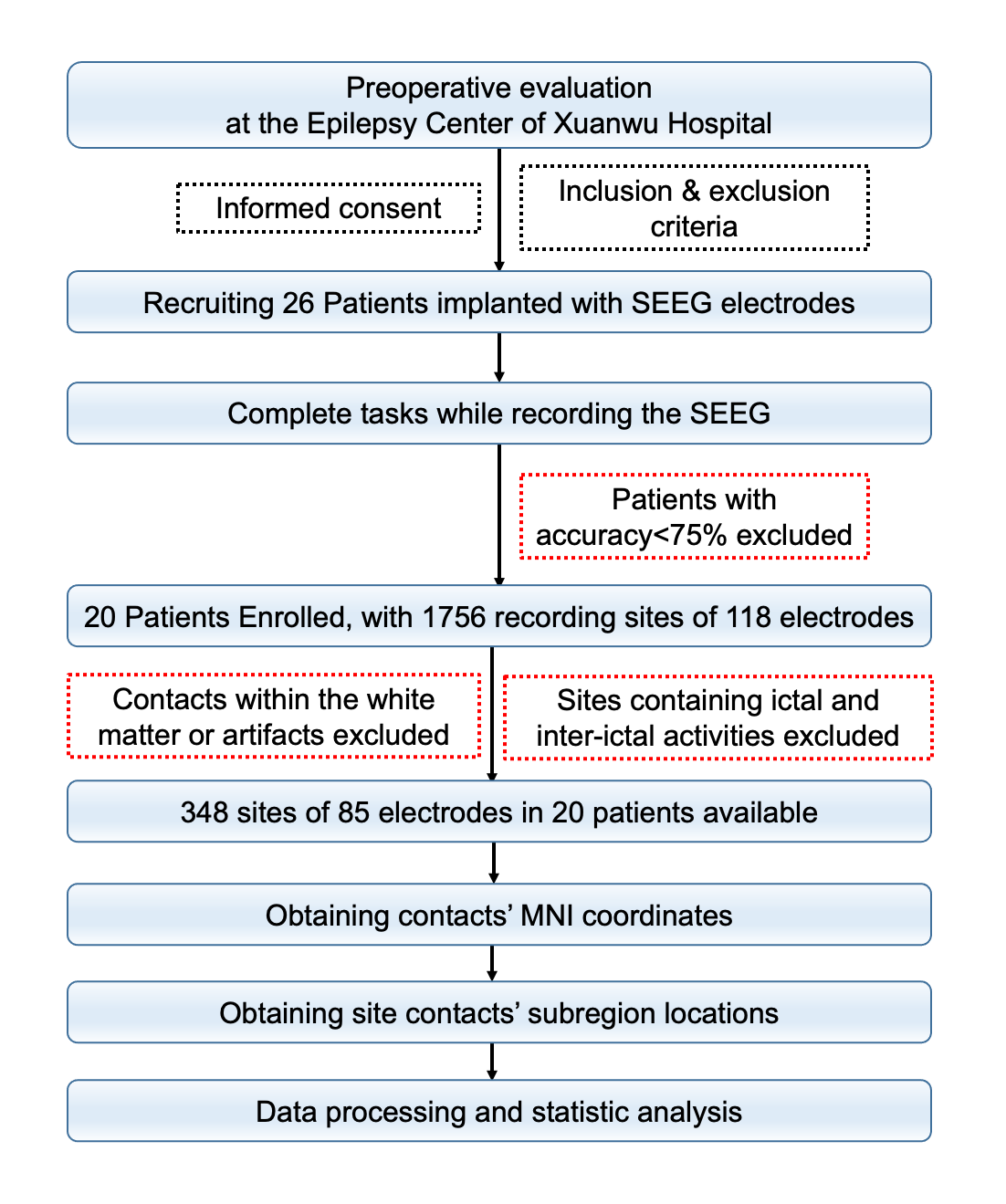
**

## **Supplemental Fig. 2: ERPs recording in the human brain.**


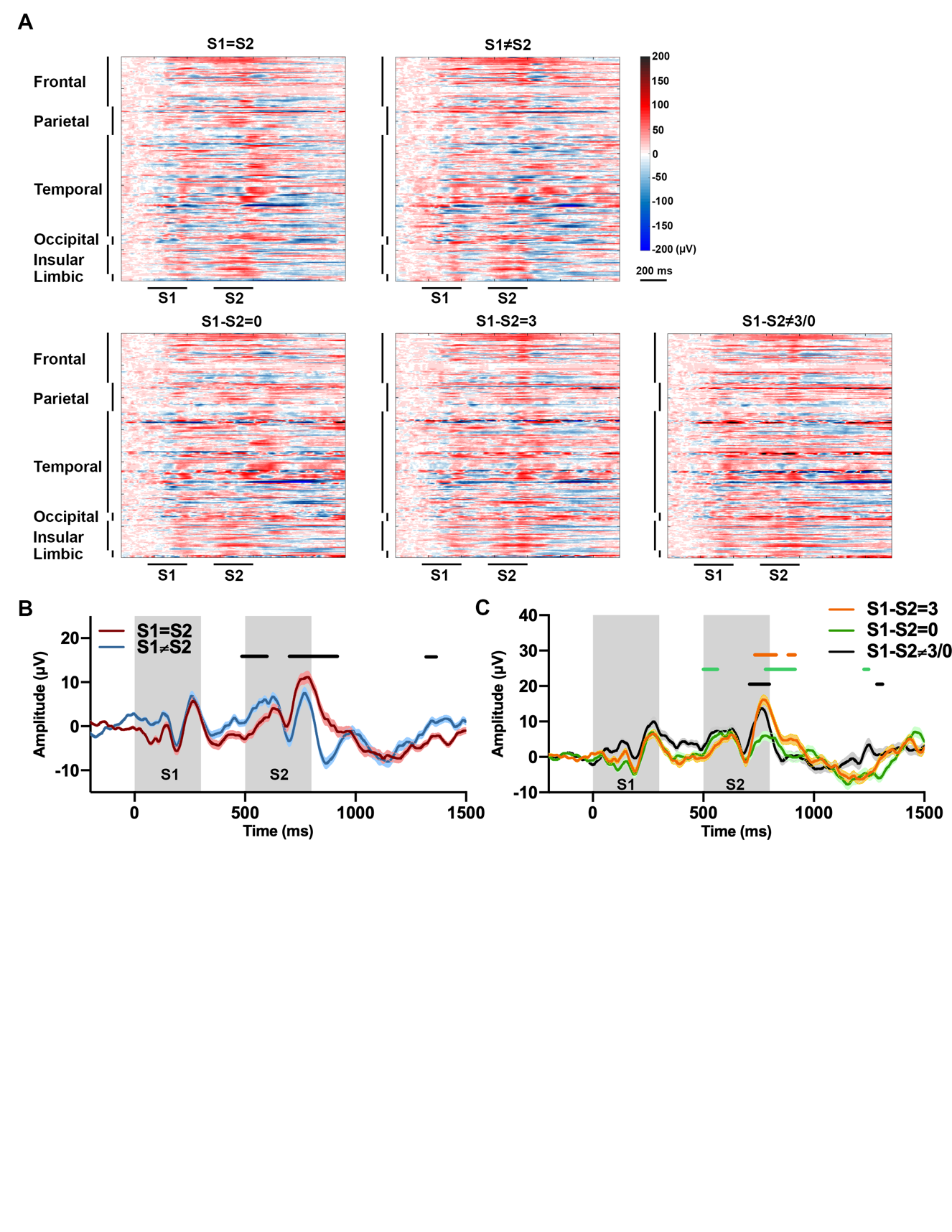


**A**, the heatmap of amplitude of 1700-ms ERPs recordings of five conditions, that is S1=S2, S1≠S2, S1-S2=0, S1-S2=3, and S1-S2≠3/0. Each row represented one recording site. The 348 sites were reorganized as the cortical lobes distributions (vertical black bar). The S1 and S2 duration were indicated as the horizontal black bar. Time scale bar as 200 ms.

**B**, the average ERPs amplitudes of total 348 electrodes of condition i (red line) and ii (blue line), with the shadow as the SEM, respectively. The grey rectangle indicated the durations of S1 and S2. The black bar presented the t-test between condition i and ii with p<0.01 over lasting for 20 ms.

**C**, the average ERPs amplitudes of total 348 electrodes of condition iii (orange line), iv (green line) and v (black line), with the shadow as the SEM, respectively. The grey rectangle indicated the durations of S1 and S2. The orange bar presented the t-test between condition iii and iv with p<0.01 over lasting for 20 ms, the green bar presented the t-test between condition iii and v with p<0.01 over lasting for 20 ms, and the black bar presented the t-test between condition iv and v with p<0.01 over lasting for 20 ms.

## **Supplemental Fig. 3: Cortical HFOs between Task 1 and 2 in five core regions.**


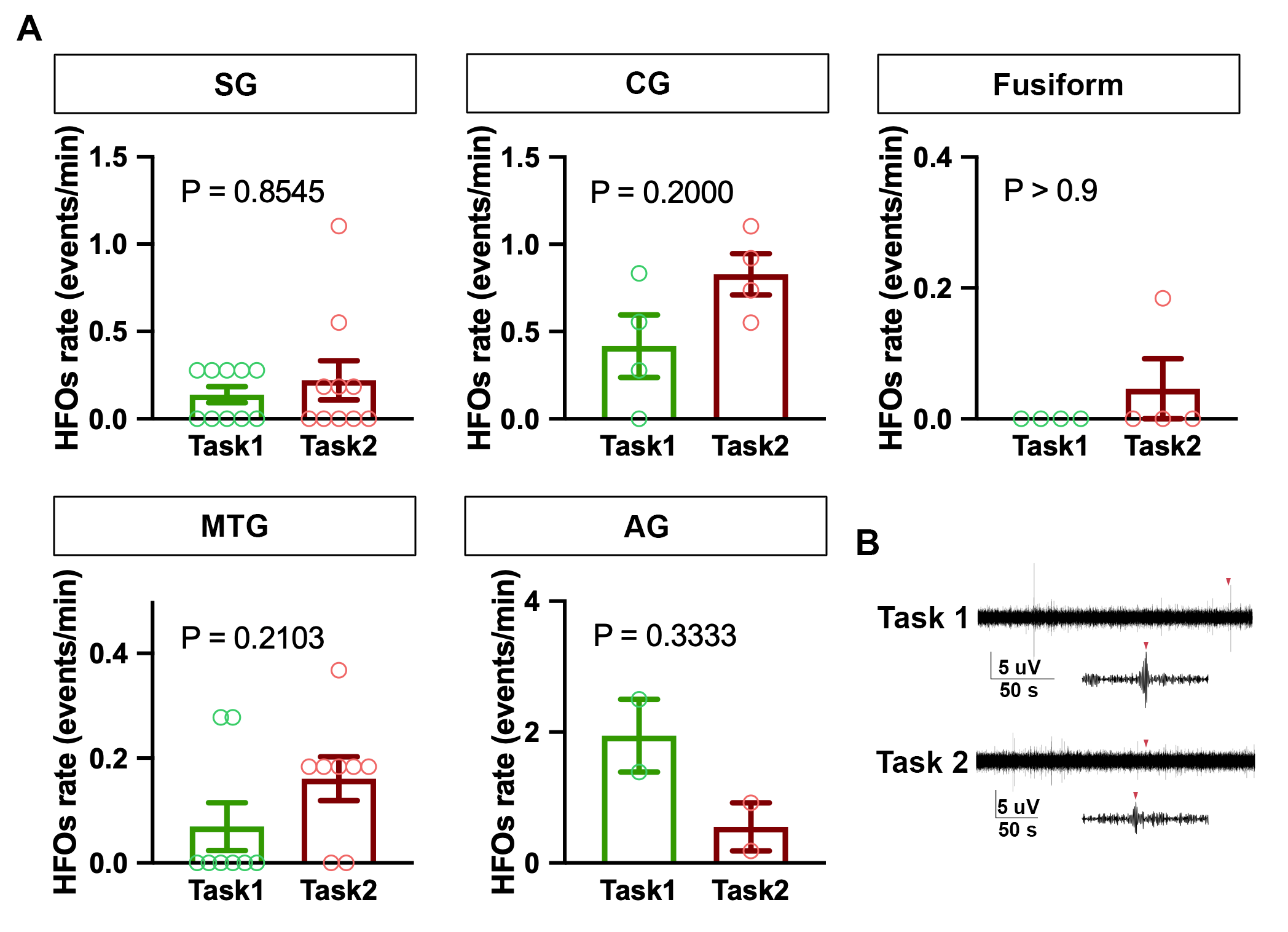


**A**, the rates of automatic detected high frequency oscillations (HFOs) in the recording of Task 1 and Task 2, in five core regions, including the SG, CG, fusiform, MTG, and AG, respectively. The rate was calculated as the ratio of the detectable event number by recording duration, which was total 216 s for Task 1 and 326 s for Task 2, respectively. Plots indicated the average rate in each task block of each sites. The Mann-Whitney test was used to compare the rate between two tasks with the P value.

**B**, representative SEEG recording of HFOs in Task 1 and 2 in the CG, with bandpass filter at 91-200 Hz. The red triangles indicated the location of the automatic detected HFO event in the recording, and the zoomed 1-s HFO event.
